# AttenCRF-U: Joint Detection of Sleep-Disordered Breathing and Leg Movements in OSA Patients

**DOI:** 10.3390/bioengineering12060571

**Published:** 2025-05-26

**Authors:** Qiuyue Li, Kewei Li, Cong Fu, Yiyuan Zhang, Huan Yu, Chen Chen, Wei Chen

**Affiliations:** 1 School of Information Science and Technology, Fudan University, Shanghai 200433, China; 2 School of Stomatology, Fudan University, Shanghai 200032, China; 3 Huashan Hospital, Shanghai Medical College, Fudan University, Shanghai 200040, China; 4 Center for Medical Research and Innovation, Shanghai Pudong Hospital, Human Phenome Institute, Fudan University, Shanghai 201203, China; chenchen_fd@fudan.edu.cn; 5 School of Biomedical Engineering, The University of Sydney, Camperdown, NSW 2006, Australia

**Keywords:** obstructive sleep apnea, sleep-disordered breathing, periodic limb movements during sleep, joint sleep event detection

## Abstract

Obstructive sleep apnea (OSA) is characterized by frequent episodes of sleep-disordered breathing (SDB), which are often accompanied by leg movement (LM) events, especially periodic limb movements during sleep (PLMS). Traditional single-event detection methods often overlook the dynamic interactions between SDB and LM, failing to capture their temporal overlap and differences in duration. To address this, we propose Attention-enhanced CRF with U-Net (AttenCRF-U), a novel joint detection framework that integrates multi-head self-attention (MHSA) within an encoder–decoder architecture to model long-range dependencies between overlapping events and employs multi-scale convolutional encoding to extract discriminative features across different temporal scales. The model further incorporates a conditional random field (CRF) to refine event boundaries and enhance temporal continuity. Evaluated on clinical PSG recordings from 125 OSA patients, the model with CRF improved the average F1 score from 0.782 to 0.788 and reduced temporal alignment errors compared with CRF-free baselines. The joint detection strategy distinguished respiratory-related leg movements (RRLMs) from PLMS, boosting the PLMS detection F1 score from 0.756 to 0.778 and the SDB detection F1 score from 0.709 to 0.728. By integrating MHSA into a CRF-augmented U-Net framework and enabling joint detection of multiple event types, this study presents a novel approach to modeling temporal dependencies and event co-occurrence patterns in sleep disorder diagnosis.

## 1. Introduction

Obstructive sleep apnea (OSA) is a common sleep disorder primarily characterized by snoring, sleep fragmentation, and frequent nocturnal awakenings. OSA impairs sleep quality and is closely associated with a range of serious health conditions, including hypertension, coronary artery disease, pulmonary heart disease, and stroke [1]. The core feature of OSA is the frequent occurrence of sleep-disordered breathing (SDB) events, including apnea and hypopnea, which are characterized by intermittent complete or partial airway collapse [2]. A key metric used to assess the severity of OSA is the apnea–hypopnea index (AHI), defined as the number of SDB events per hour of sleep. According to the AHI classification criteria, OSA can be classified as mild, moderate, or severe [3]. Accurate detection of SDB events is essential for the clinical assessment of OSA, as AHI directly influences decisions regarding treatment interventions, such as continuous positive airway pressure (CPAP) [3].

In OSA patients, SDB events often co-occur with leg movements (LM). LM typically manifests as involuntary muscle contractions, commonly observed in the toes, ankles, knees, and hips, primarily involving contractions of the tibialis anterior muscle [4]. When LM occurs in a periodic manner, it is referred to as periodic limb movements during sleep (PLMS), which may disrupt sleep architecture, leading to sleep fragmentation and daytime functional impairments [5]. Although patients are often unaware of the presence of PLMS, it can lead to difficulties falling asleep, excessive daytime sleepiness, reduced attention, and decreased work efficiency [6]. A key parameter for evaluating PLMS is the PLMS index (PLMI), which quantifies the number of PLMS events per hour. OSA patients with comorbid PLMS exhibit worse disease outcomes and daytime symptoms, including a higher risk of adverse cardiovascular events [7]. Epidemiological data show that 14.8% of OSA patients have a PLMI exceeding 15/h, underscoring the clinical importance of PLMS in OSA progression [8].

In current clinical practice, the annotation of sleep events is primarily based on whole-night polysomnography (PSG), with manual scoring performed by specialists according to the American Academy of Sleep Medicine (AASM) guidelines. PSG includes multiple physiological signals such as electroencephalogram (EEG), electrooculogram (EOG), electromyogram (EMG), and electrocardiogram (ECG) [9].

SDB events include apnea, defined as a ≥90% airflow reduction lasting ≥10 s during sleep, and hypopnea, defined as a ≥30% reduction for ≥10 s, accompanied by either a ≥3% oxygen desaturation or an arousal [10].

LM events are annotated using tibialis anterior EMG signals, with the onset marked by an amplitude >8 μV above the baseline and offset as a sustained drop <2 μV for ≥0.5 s, with durations ranging from 0.5 to 10 s. A PLMS series is identified when ≥4 LMs occur with inter-movement intervals of 5–90 s [11].

SDB events can elicit respiratory-related leg movements (RRLMs), defined as LMs occurring within 0.5 s before to 0.5 s after an SDB event. RRLMs should be excluded from PLMS annotations to prevent misclassification [11].

Although manual scoring remains the clinical gold standard, it imposes a significant clinical burden, including an average of 1–3 h of labor per PSG recording for expert annotation and inter-rater variability rates of 15–20% in event classification [9]. Extensive research has been conducted on single-event automatic detection, including studies targeting LM [12,13] and SDB [14,15,16].

To meet the growing clinical need for comprehensive evaluation, the joint detection of multiple sleep events has become a key research direction, offering richer diagnostic insights and supporting personalized interventions. Furthermore, LM and SDB events frequently coexist in the same patient, especially among those with OSA, where LM incidence is significantly higher and closely related to sleep quality deterioration caused by OSA [17].

Therefore, previous works have leveraged multi-modal physiological signals and various modeling strategies for joint LM and SDB detection. For instance, Waltisberg et al. [18] combined minimum redundancy maximum relevance (mRMR) for feature selection with a Bayesian classifier to classify 60-s segments into normal, LM, and SDB events. Biswal et al. [19] employed a recursive convolutional neural network (CNN) for sleep staging and LM/SDB recognition. They divided events into 1 s segments for classification across large-scale PSG datasets. Zahid et al. [20] proposed a Multi-Modal Sleep Event Detection (MSED) model that combines a CNN, bidirectional gated recurrent unit (biGRU), and attention mechanism to extract event features through multiple processing streams. Their model outperformed single-event detection models. The DeepSDBPLM model designed by Almutairi et al. [21] integrates empirical mode decomposition with attention for the joint classification of SDB and PLM events within 30 s signal segments. Despite these advances, most existing methods still rely on fixed-time windows, limiting temporal resolution and clinical applicability. To overcome this, recent studies have shifted towards event-based modeling. Seeuws et al. [22] proposed learning directly from event annotations without post-processing, and later introduced a human-in-the-loop annotation framework [23] to improve adaptability with minimal manual effort.

The key challenges of joint detection lie in the temporal overlap between LM and SDB events, as well as their differing durations, both of which require models capable of capturing fine-grained and long-range temporal dependencies. Moreover, engineering constraints such as multi-channel signal synchronization and limitations of conventional acquisition systems pose barriers to scalable deployment. To address these issues, this study proposes an automatic joint detection framework for LM and SDB events in OSA patients, aiming to enhance diagnostic reliability, reduce clinical burden, and enable future integration into wearable or bedside monitoring systems.

This study makes three key contributions. First, a joint detection strategy is proposed to reduce isolated event errors by reinforcing the associations between LM and SDB events, enabling accurate recognition of RRLM and PLMS series. Second, temporal dependency is enhanced through multi-head self-attention (MHSA) to capture long-range temporal relationships, while a conditional random field (CRF) refines prediction continuity to reduce fragmentation in event sequences. Third, an encoder–decoder structure with multi-scale convolutional operations is designed to extract discriminative patterns across different temporal scales, improving detection granularity and adapting to heterogeneous event durations.

## 2. Materials and Methods

### 2.1. Datasets

Data collection was conducted at Huashan Hospital from August 2021 to April 2024 with ethical approval (Approval No. 2021-811). All 125 adult participants provided written informed consent and were diagnosed with OSA (AHI≥5). The cohort spanned mild to severe cases and included common comorbidities such as hypertension, obesity, and insomnia (Table 1).

Whole-night PSG recordings included six EEG channels (F3/M2, F4/M1, C3/M2, C4/M1, O1/M2, O2/M1), three chin EMG channels (Chin1, Chin2, Chin3), one ECG channel (ECG), two EOG channels (E1, E2), left and right tibialis anterior EMG channels (Leg/L and Leg/R), and multiple respiratory signals, including nasal airflow pressure (Nasal), oxygen saturation, thoracic effort (Thor), and abdominal effort (Abdo). All signals were sampled at 1024 Hz. Patients refrained from taking LM-affecting medications for at least 2 weeks prior to the recording. Events were re-annotated by trained technicians following AASM guidelines, covering LM, PLMS, apnea, and hypopnea.

**Figure 1:**
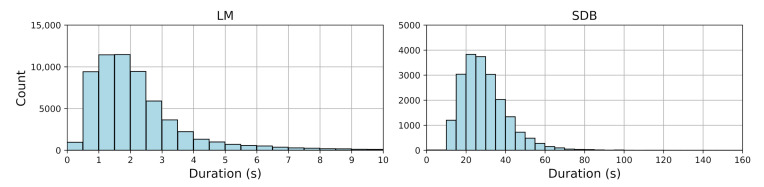
Histogram of LM and SDB event durations.

Only sleep epochs were included. Figure 1 shows the duration distribution of LM and SDB events. Table 2 provides descriptive statistics, including event counts, median durations with interquartile ranges (IQRs), and mean durations with standard deviations and ranges for both LM and SDB events. LM events mostly ranged from 0.5 to 2.5 s, indicating a clear clustering pattern, while SDB events exhibited greater variability (10–151.52 s), reflecting their complexity.

### 2.2. Overview of AttenCRF-U (Attention-Enhanced CRF with U-Net)

**Figure 2:**
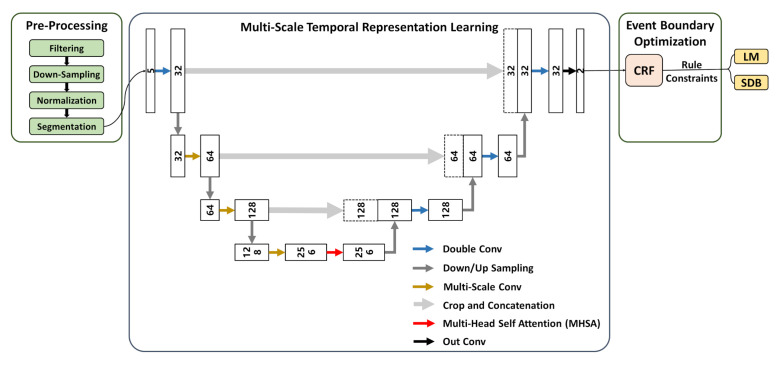
Overview of AttenCRF-U for detecting LM and SDB.

The framework consists of three major components, as illustrated in the following Figure 2:

Data pre-processing. Raw signals are filtered, down-sampled, normalized, and segmented to improve signal quality and remove noise, resulting in reliable input.

Multi-scale temporal representation learning. A multi-scale deep learning architecture is employed to enhance the representation of event features across varying temporal scales. Specifically, the U-Net backbone improves temporal resolution through an encoder–decoder structure. MHSA captures long-range dependencies and strengthens global semantic perception. Multi-scale convolution adapts to both short- and long-duration events using receptive fields of different sizes, which improves the recognition of diverse event types.

Event boundary optimization. CRF is introduced to refine event boundaries, enhancing continuity and consistency in event sequences. Physiology-based rule constraints are incorporated to filter out implausible predictions, thereby enhancing the medical interpretability of detection results.

### 2.3. Data Pre-Processing

Five PSG signal channels were selected as input. The Leg/L and Leg/R channels are used to detect LM events, while the Abdo, Thor, and Nasal channels are used as the reference channels for SDB events. The pre-processing pipeline consists of filtering, down-sampling, normalization, and segmentation. The filtering process includes the use of a fourth-order Butterworth band-pass filter with cutoff frequencies of 10 Hz and 50 Hz for the EMG signals [10]. For the thoracic effort and abdominal effort signals, second-order Butterworth band-pass filters with cutoff frequencies of 0.1 Hz and 15 Hz are applied to emphasize respiratory frequency characteristics. The nasal airflow pressure signal is filtered within the range of 0.03 Hz to 50 Hz to eliminate low-frequency drift and high-frequency noise [10]. All channels are down-sampled to 100 Hz to ensure consistency and comparability across signals. Z-score normalization is applied individually to each channel for each patient. The normalization is performed on the time sequence $x_{1},x_{2},\ldots ,x_{N}$, and is defined as

(1a) $$\begin{array}{cc} y_{i} & =\frac{x_{i}-\bar{x}}{s} \end{array}$$

(1b) $$\begin{array}{cc} \bar{x} & =\frac{1}{N}\sum_{i=1}^{N}x_{i} \end{array}$$

(1c) $$\begin{array}{cc} s & =\sqrt{\frac{1}{N-1}\sum_{i=1}^{N}{\left(x_{i}-\bar{x}\right)}^{2}} \end{array}$$

The signal is segmented into 60 s epochs, offering a trade-off between temporal granularity and global contextual awareness. To ensure model relevance, the framework is trained only on segments containing at least one event. To enable precise point-wise detection and account for potential temporal overlaps between LM and SDB events, event labels are generated by aligning manually annotated event intervals with the sampling resolution of the signals to produce point-wise label sequences. No event prioritization is applied during this process, and all annotated events are retained. Each time point is assigned a two-dimensional label vector [LM_label, SDB_label], where each element is either 1 (presence) or 0 (absence).

### 2.4. Multi-Scale Temporal Representation Learning

This module adopts an encoder–decoder structure that strengthens temporal representation through a hierarchical structure: the encoder compresses input to extract high-level features, while the decoder restores fine-grained details to ensure accurate boundary reconstruction. Skip connections are used to fuse shallow local features with deep global semantics, effectively reducing information loss [24].

(1) Multi-Scale Conv

**Figure 3:**
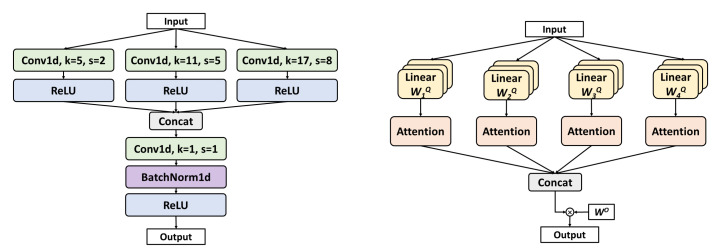
Implementation details of Multi-Scale Conv and MHSA.

Each encoder layer employs parallel multi-scale convolution, as shown in Figure 3 (left). The kernel sizes (5, 11, 17) are selected to capture short, medium, and long temporal patterns to match the durations of LM (0.5–10 s) and SDB (>10 s) events. Smaller kernels are better at detecting transient EMG bursts (LM), while larger kernels capture sustained respiratory patterns (SDB).

(2) MHSA

The bottleneck layer incorporates MHSA, a mechanism originally proposed by Vaswani et al. [25], as shown in Figure 3 (right). Four self-attention heads are employed. The computation of MHSA proceeds as follows:

The input is linearly projected into query (*Q*), key (*K*), and value (*V*) vectors using head-specific weights $W_{i}^{Q}$, $W_{i}^{K}$, and $W_{i}^{V}$ for each attention head, as follows:

(2a) $$\begin{array}{cc} Q_{i} & =QW_{i}^{Q} \end{array}$$

(2b) $$\begin{array}{cc} K_{i} & =KW_{i}^{K} \end{array}$$

(2c) $$\begin{array}{cc} V_{i} & =VW_{i}^{V} \end{array}$$

where i=1,2,…,h, and *h* is the number of heads. Each head computes its respective attention weights according to the following formula:

(3) $${\mathrm{Attention}}_{i}=\mathrm{softmax}\left(\frac{Q_{i}K_{i}^{T}}{\sqrt{d_{k}}}\right)V_{i}$$

where $d_{k}$ is the dimensionality of the *K* vectors, and $\sqrt{d_{k}}$ serves as a scaling factor. Finally, the outputs from all attention heads are concatenated and passed through a linear transformation using a weight matrix $W^{O}$ to produce the final output, as follows:

(4) $$\mathrm{Output}=\mathrm{Concat}\left({\mathrm{Attention}}_{1},\ldots ,{\mathrm{Attention}}_{h}\right)W^{O}$$

MHSA offers two key benefits: it captures multi-scale temporal dependencies, facilitating the extraction of features from LM and SDB events of varying durations; and it enables efficient parallel processing of long sequences (e.g., 60 s), outperforming traditional methods such as long short-term memory (LSTM) in both accuracy and computational speed.

### 2.5. Event Boundary Optimization

Point-wise predictions from temporal representation learning may result in short, isolated events or imprecise boundaries, compromising detection consistency. To address this issue, an optimization module that combines CRF [26] with rule-based constraints is introduced. The CRF captures temporal dependencies to globally refine event boundaries, reducing fragmentation and boundary shifts. It smooths predictions, enforces continuity at event onsets and offsets, and eliminates implausible patterns (e.g., rapid LM fluctuations), thus ensuring physiological plausibility [27,28]. The CRF workflow is presented in Algorithm 1.
**Algorithm 1:** CRF-Based Temporal Smoothing for LM and SDB Detection**Require:** 
Predicted probabilities ${\left\{P_{i}^{e}\right\}}_{i=1}^{T}$, features ${\left\{f_{i}=\left(t_{i},c_{i}\right)\right\}}_{i=1}^{T}$; number of iterations N=5**Ensure:** 
Smoothed labels ${\left\{x_{i}^{e}\right\}}_{i=1}^{T}$, for e∈{LM,SDB}1:**for** each e∈{LM,SDB} **do**2:    Compute unary potential: $\psi_{u}^{e}\left(x_{i}\right)=-\log P_{i}^{e}$3:    Define pairwise potential: $\psi_{p}^{e}\left(x_{i},x_{j}\right)=\mu \left(x_{i},x_{j}\right)\sum_{m=1}^{2}\omega^{m}K^{m}\left(f_{i},f_{j}\right)$4:    where$$\mu \left(x_{i},x_{j}\right)=\left\{\begin{array}{cc} 1, & \mathrm{if}\;x_{i}\neq x_{j}\\ 0, & \mathrm{else} \end{array}\right.$$$$K^{1}\left(f_{i},f_{j}\right)=\exp\left(-\frac{∥t_{i}-t_{j}{∥}^{2}}{2\theta_{\alpha }^{2}}-\frac{∥c_{i}-c_{j}{∥}^{2}}{2\theta_{\beta }^{2}}\right),K^{2}\left(f_{i},f_{j}\right)=\exp\left(-\frac{∥t_{i}-t_{j}{∥}^{2}}{2\theta_{\gamma }^{2}}\right)$$5:    Initialize marginal: $Q_{i}^{e}\left(x\right)\propto \exp\left(-\psi_{u}^{e}\left(x_{i}=x\right)\right)$6:    **for** k=1 to *N*                                                  ▹ Iterative update for N=5 steps **do**7:        **for** each *i* **do**8:               Compute message: $m_{i}^{e}\left(x\right)=\sum_{j\neq i}\sum_{m=1}^{2}\omega^{m}K^{m}\left(f_{i},f_{j}\right)Q_{j}^{e}\left(x\right)$9:               Update marginal: $Q_{i}^{e}\left(x\right)\propto \exp\left(-\psi_{u}^{e}\left(x_{i}=x\right)-m_{i}^{e}\left(x\right)\right)$10:        **end for**11:    **end for**12:    Final label: $x_{i}^{e}=\arg\max_{x}Q_{i}^{e}\left(x\right)$13:**end for**

Rule-based constraints, guided by AASM guidelines, further refine predictions. Adjacent LM events less than 0.5 s apart are merged, and thresholding removes events that are too short or excessively long, reducing false detections due to noise or abnormal patterns. Together, CRF and rule-based refinement yield temporally coherent outputs that better align with true event boundaries, enhancing both prediction consistency and clinical interpretability.

### 2.6. Experimental Settings and Evaluation Metrics

Patients were randomly split in a 9:1 ratio, and only segments with stable signal quality and complete annotations were used. Experiments were conducted on a Linux system equipped with an Intel® Xeon® Gold 5218 CPU (Intel Corporation, Santa Clara, CA, USA) (2.30 GHz) and two NVIDIA GeForce RTX 3090 GPUs (NVIDIA Corporation, Santa Clara, CA, USA). The models were implemented in Python 3.8.12 with PyTorch 1.10.1. The framework was trained using the Adam optimizer [29] with a learning rate of 0.001 and a weight decay of 0.0001, over 50 epochs with a batch size of 128. The loss function was the average Dice loss across all classes, as defined in Equation (5), which is suitable for multi-class outputs. Compared with cross-entropy, Dice loss more effectively addresses class imbalance, particularly when foreground events (e.g., LM and SDB) are sparse [30,31].

(5) $$\mathrm{Dice}\;\mathrm{Loss}=1-\frac{1}{C}\sum_{i=1}^{C}\frac{2|X_{i}\cap Y_{i}|+10^{-3}}{|X_{i}\left|+\right|Y_{i}|+10^{-3}}$$

where $X_{i}$ and $Y_{i}$ denote the predicted and ground truth labels for class *i*, respectively. A Laplace smoothing term of $10^{-3}$ is added to prevent division by zero [31].

The detection framework is evaluated based on point-wise predictions, comparing the output with ground truth labels at each time point. The performance for LM and SDB event detection is assessed using the following metrics:

(6) $${\mathrm{Precision}}_{i}=\frac{{\mathrm{TP}}_{i}}{{\mathrm{TP}}_{i}+{\mathrm{FP}}_{i}}$$

(7) $${\mathrm{Recall}}_{i}=\frac{{\mathrm{TP}}_{i}}{{\mathrm{TP}}_{i}+{\mathrm{FN}}_{i}}$$

(8) $$F1\;{\mathrm{Score}}_{i}=\frac{2\cdot {\mathrm{Precision}}_{i}\cdot {\mathrm{Recall}}_{i}}{{\mathrm{Precision}}_{i}+{\mathrm{Recall}}_{i}}$$

(9) $$\mathrm{Average}\;F1\;\mathrm{Score}=\frac{1}{C}\sum_{i=1}^{C}F1\;{\mathrm{Score}}_{i}$$

where *C* denotes the number of classes, and ${\mathrm{TP}}_{i}$, ${\mathrm{FP}}_{i}$, and ${\mathrm{FN}}_{i}$ represent the true positives, false positives, and false negatives for class *i*, respectively.

For a more comprehensive assessment, event-level evaluation is introduced, treating entire events as the evaluation unit, unlike point-wise metrics. The match between predicted and ground truth events is determined using the Intersection over Union (IoU) [32]. A predicted event is considered correctly detected if its IoU with the corresponding ground truth event exceeds a threshold of 0.3. The IoU is defined as

(10) $$\mathrm{IoU}=\frac{\left|X\cap Y\right|}{\left|X\cup Y\right|}$$

where *X* and *Y* are the temporal intervals of the predicted and ground truth events, respectively. In addition to standard event-level precision, recall, and F1 score, the evaluation includes temporal alignment errors to quantify accuracy in locating event boundaries. These errors consist of onset error, offset error, and duration error, defined as the difference between predicted and actual time points. A positive value indicates a delayed prediction, while a negative value indicates an early prediction.

## 3. Results

**Figure A1:**
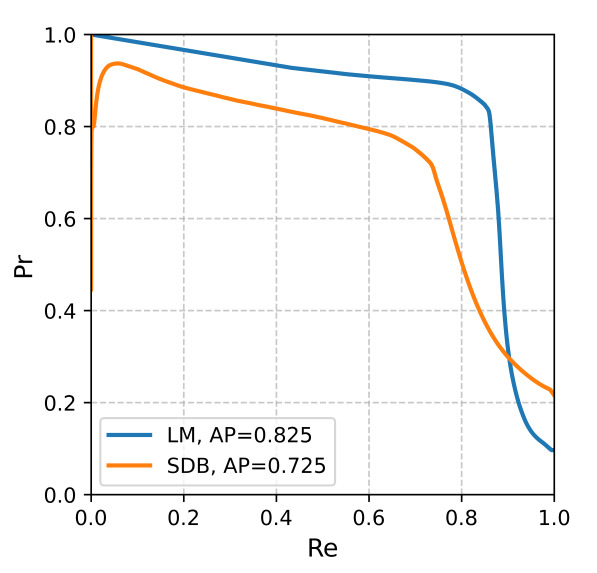
Precision–recall curves for LM and SDB event detection.

The performance of the proposed AttenCRF-U framework was systematically evaluated on the Huashan dataset for both LM and SDB event detection (Table 3). For LM events, the F1 score was 0.848, with a precision of 0.862 and a recall of 0.834, indicating strong stability and accuracy. For SDB events, the F1 score of 0.728 was lower than that of LM, which can be attributed to two primary factors. First, SDB events are less frequent, while Dice loss assigns equal weight across classes. Moreover, SDB events often exhibit subtle and gradual onset/offset transitions, making their boundaries more difficult to localize than the abrupt EMG bursts characteristic of LM. The overall average F1 score reached 0.788, demonstrating robust and balanced detection performance across event categories. Precision–recall curves (Figure A1) show that LM and SDB events achieved average precision (AP) values of 0.825 and 0.725, respectively.

Further event-level evaluation results are summarized in Table 4. With the IoU threshold set at 0.3, the F1 scores for LM and SDB events improved to 0.863 and 0.739, respectively, which are higher than the point-wise results, indicating more temporally coherent predictions at the event level. In terms of temporal alignment error, LM events showed a slight onset error of 0.034 s, an offset error of −0.051 s, and a duration error of −0.085 s, suggesting a conservative tendency. In contrast, SDB events exhibited more pronounced boundary errors, with an onset error of 2.264 s, an offset error of −2.202 s, and a duration error of −4.466 s. This is likely due to the longer duration and more variable patterns of SDB events. Although a lower IoU threshold increases tolerance and reduces false negatives, it can also compromise boundary precision, which affects longer events like SDB more evidently.

**Figure 4:**
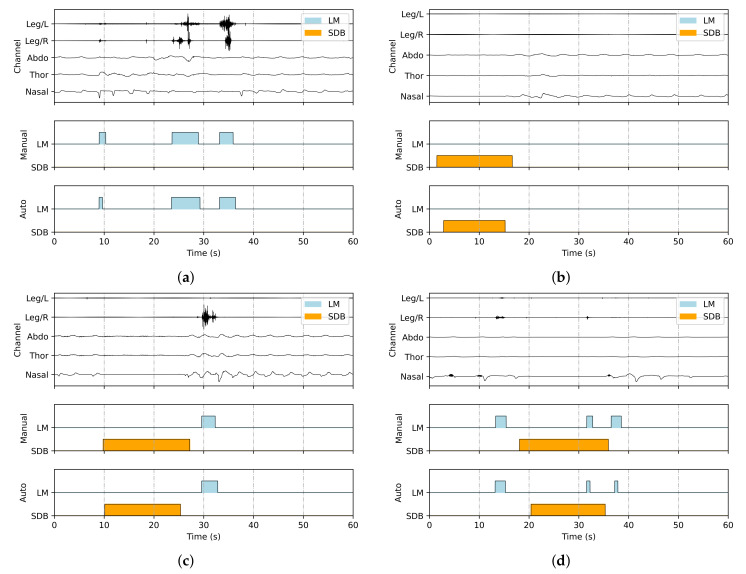
Representative visualizations of joint detection results, with LM and SDB events marked in blue and orange, respectively: (**a**) only LM events, (**b**) only SDB events, (**c**) co-occurrence of LM and SDB events without overlap, and (**d**) co-occurrence of LM and SDB events with overlap.

Figure 4 visualizes annotated signal segments, showing all five input channels (Leg/L, Leg/R, Abdo, Thor, and Nasal), along with manual and automatic annotations of LM and SDB events. The predictions align closely with the ground truth, especially in onset and offset timing. Furthermore, the framework effectively distinguishes overlapping events, ensuring both accurate classification and precise duration estimation.

## 4. Discussion

### 4.1. Comparative Analysis of the Effect of Multi-Scale Temporal Representation Learning

**Figure 5:**
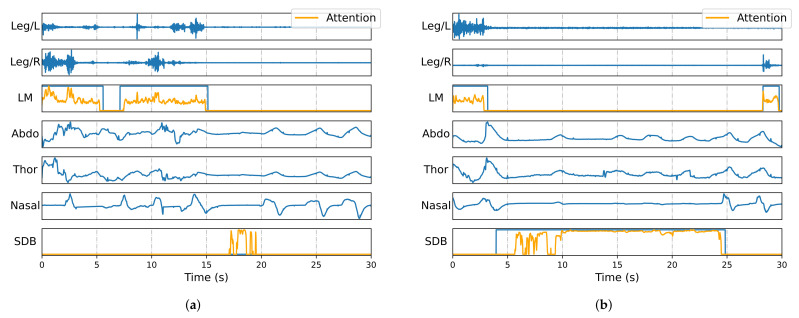
Grad-CAM-based attention visualization of PSG signal model for LM and SDB event detection, where orange represents attention values, and blue represents different signals and predicted events: (**a**) example 1 and (**b**) example 2.

To illustrate how the attention module attends to relevant temporal segments when identifying events, gradient-weighted class activation mapping (Grad-CAM) [33] was employed for interpretability analysis of the trained model. Grad-CAM generated attention heatmaps for the two event classes, with values exceeding 1 truncated to 1, and overlaid them onto the original signals and labels for visualization. These visualizations help interpret the features that most contribute to each class of events and reveal different attention patterns. As shown in Figure 5, the attention values of the framework increase significantly in the temporal locations corresponding to event occurrences, indicating that the model effectively focuses on the signal regions associated with the target events.

An ablation study (Table A1) compared convolutional kernel combinations (5, 11, 17). Single-kernel performance degraded with increasing size, as smaller kernels excelled at capturing boundary details. The triple-kernel setup achieved the best avg F1 (0.788), confirming that multi-scale fusion is critical for detecting LM/SDB patterns.

### 4.2. Comparative Analysis of the Effect of Event Boundary Optimization

**Figure 6:**
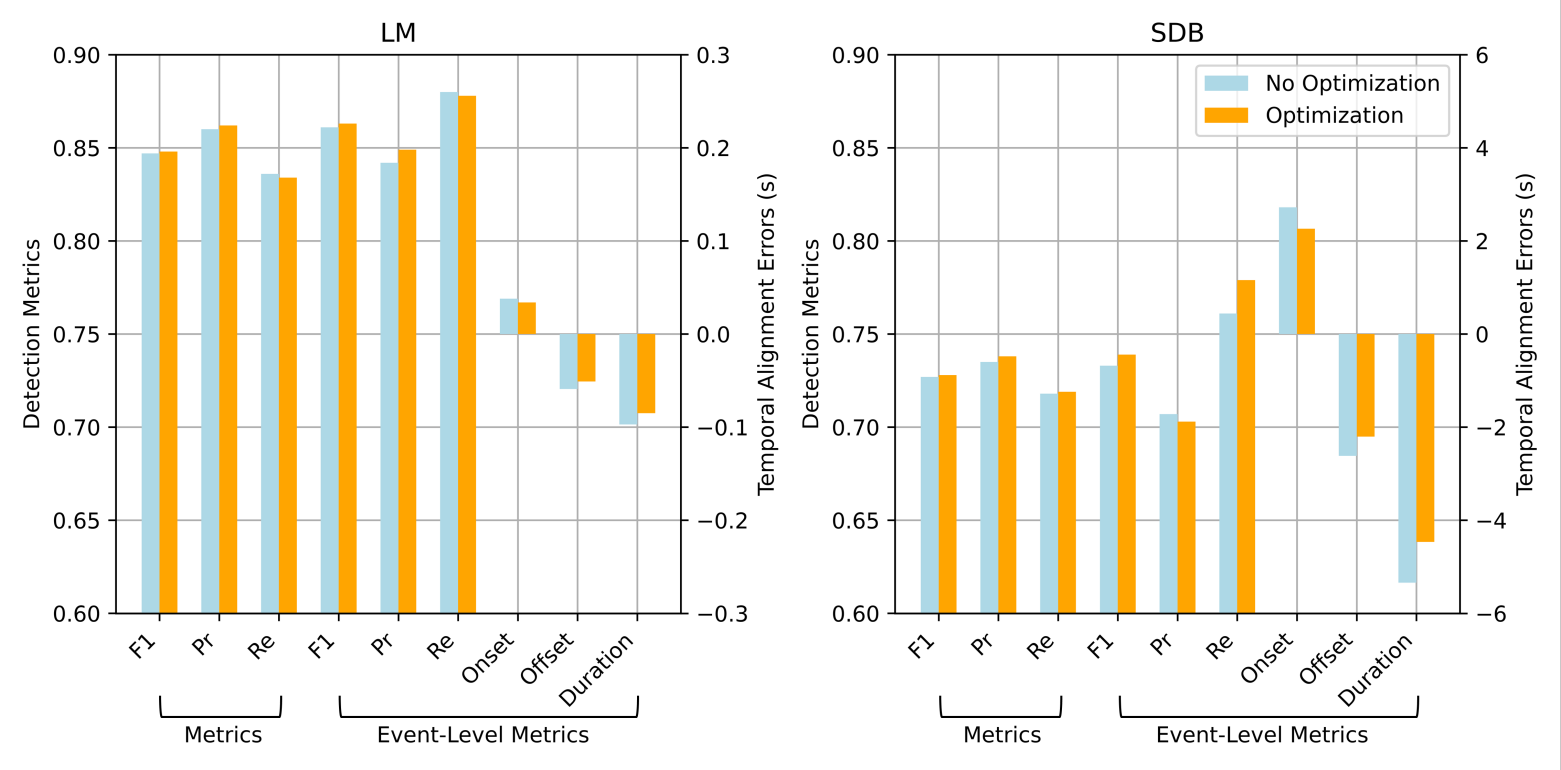
Comparison of detection performance and temporal alignment errors for LM and SDB events with and without event boundary optimization.

Key metrics before and after event boundary optimization were compared to assess the effect of this module. As shown in Figure 6, although the overall improvements are modest, it consistently enhances precision and temporal alignment.

For LM events, the point-wise F1 score improved slightly from 0.847 to 0.848, with precision increasing from 0.860 to 0.862 and a minor drop in recall. At the event level, the F1 score increased from 0.861 to 0.863, precision changed from 0.842 to 0.849, and recall slightly decreased to 0.878. This suggests that event boundary optimization reduces false positives by refining boundary estimates. Moreover, the onset and offset errors decreased from 0.038 s to 0.034 s and from −0.059 s to −0.051 s, respectively, indicating improved temporal alignment with the ground truth. Similar trends were observed for SDB detection. Event boundary optimization improved all major metrics and notably reduced boundary errors, demonstrating its effectiveness in handling long-duration and complex events.

### 4.3. Comparative Analysis of Single and Joint Detection Strategies for Multi-Event Detection

A comparative analysis was conducted between single and joint detection strategies for LM, PLMS, and SDB events. In the single detection strategy, the network architecture remained unchanged, but input channels and loss functions were adjusted for each event type: Leg/L and Leg/R for LM, and Abdo, Thor, and Nasal for SDB. The same training and pre-processing protocols were applied to ensure fair comparison. PLMS detection in the single strategy relied on periodicity analysis, while the joint strategy excluded RRLM based on automatically detected SDB events and identified PLMS from the remaining LM events.

**Figure 7:**
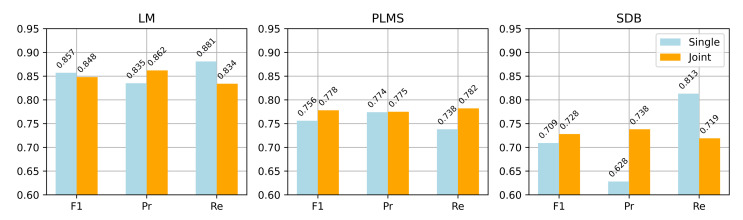
Performance comparison between single and joint detection across LM, PLM, and SDB events (point-wise F1 score, precision, and recall).

Figure 7 compares the two strategies across the three event types in terms of point-wise F1 score, precision, and recall. The joint detection strategy achieved better recall and stability for PLMS and SDB events, while maintaining strong LM detection performance. Specifically, the PLMS F1 score improved from 0.756 to 0.778, and SDB from 0.709 to 0.728, indicating enhanced generalization for complex event patterns. From a theoretical perspective, joint detection leverages shared features among related events, leading to better discrimination and aligning more closely with clinical needs for integrated multi-event analysis.

### 4.4. Performance in Comparison with Other Proposed Methods

To validate the effectiveness of the proposed AttenCRF-U framework, we compared its performance against two representative baselines: MSED [20] and Event-Based Modeling [22]. Since MSED originally includes arousal events in its detection pipeline, we modified its input configuration to include only the two relevant information streams (leg and respiratory signals), while keeping the structure unchanged. Event-Based Modeling follows the design proposed by Seeuws et al. [22], which uses a U-Net backbone to predict event centers and durations. All methods were evaluated on the Huashan dataset, with the results summarized in Table 5.

For LM event detection, AttenCRF-U achieved a substantially higher F1 score of 0.848, compared with 0.756 for Event-Based Modeling and 0.691 for MSED. Similarly, for SDB events, AttenCRF-U attained an F1 score of 0.728, outperforming Event-Based Modeling (0.705) and MSED (0.633). These results demonstrate that AttenCRF-U offers significant improvements in both event categories.

The superior performance of AttenCRF-U can be attributed to its integrated design, which combines multi-scale temporal feature extraction via U-Net, enhanced contextual representation through multi-head attention, and structured sequence inference with CRF. However, one limitation of the proposed method lies in its reliance on post-processing to further optimize event boundaries.

### 4.5. Evaluation of Detection Performance Based on Respiratory Channel Combinations

This study used three respiratory signal channels—Abdo, Thor, and Nasal—which collectively capture respiratory motion patterns and rate variability during sleep. To investigate the impact of these channels on detection performance, we designed comparative experiments using (1) single-channel input (e.g., Nasal only), (2) dual-channel combinations (e.g., Abdo + Nasal), and (3) a three-channel fusion (Abdo + Thor + Nasal). The EMG channels (Leg/L and Leg/R) were held constant across all configurations. The evaluation results are presented in Table 6, including point-wise F1 score, precision, and recall for different event types. The results indicate that the configuration of the respiratory channels significantly affects the detection of both SDB and LM events.

Among single-channel inputs, the Nasal channel achieved the highest average F1 score (0.775), with an F1 score of 0.711 for SDB events, demonstrating strong discriminative power for respiratory event detection. However, its F1 score for LM events (0.838) was not superior to other channels. In contrast, the Abdo and Thor channels yielded slightly lower average F1 scores, with SDB F1 scores of 0.631 and 0.612, respectively, but slightly better LM detection (F1 scores of 0.847 and 0.851, respectively). This may be due to subtle postural changes during LM events, which affect thoracoabdominal respiratory signals. Although these signals are not direct indicators of LM, they provide useful auxiliary cues.

In dual-channel combinations, Abdo + Thor improved the average F1 score to 0.764, with an SDB F1 score of 0.682, showing that fusing thoracoabdominal motion signals enhances respiratory anomaly detection. When Nasal was combined with either Abdo or Thor, SDB detection further improved, with F1 scores of 0.718 and 0.706, respectively, while LM detection remained stable (F1 scores of 0.839 and 0.848). This indicates a complementary effect between airflow and respiratory motion signals, enhancing the ability to identify SDB events.

The three-channel fusion configuration (Abdo + Thor + Nasal) achieved the best overall performance, with the highest average F1 score (0.788). SDB event detection reached its peak F1 score of 0.728, while LM detection remained robust (F1 score = 0.848). These results demonstrate that multi-channel integration maximizes the advantages of individual signals, improving detection accuracy and stability.

Notably, SDB detection is primarily driven by the Nasal channel. Even in the Nasal-only setting, its F1 score (0.711) closely approached the optimal performance achieved with the three-channel combination (0.728). This can be attributed to the fact that nasal airflow directly reflects respiratory flow and aligns well with manual annotations, making it more sensitive to apnea and hypopnea events than the Abdo and Thor channels. When Abdo and Thor signals are incorporated, the model benefits from additional information on chest and abdominal movements, which helps capture diverse respiratory patterns and improves robustness under signal variability or partial signal loss.

Although respiratory signals do not directly capture limb activity, LM detection is influenced by their choice. The Thor-only configuration achieved the highest F1 score (0.851) for LM detection, likely due to physiological coupling and signal crosstalk. LMs, especially periodic ones, often involve subtle postural shifts or respiratory modulation, leading to correlated thoracic movements. Moreover, muscle crosstalk may produce detectable low-frequency changes in thoracic effort signals. In contrast, abdominal and nasal channels provide limited relevance. Specifically, abdominal movements are more closely associated with breathing effort rather than limb activity, while nasal airflow often remains stable during LM. Including these channels may introduce noise that masks the informative Thor patterns. This observation highlights the need for further investigation using more precise biomechanical data.

From a signal acquisition perspective, the Nasal channel uses a nasal cannula connected to a pressure sensor to estimate airflow via pressure fluctuations, offering high sensitivity for SDB detection [34]. However, it may interfere with natural sleep and reduce user comfort. In comparison, thoracoabdominal signals are collected via respiratory inductive plethysmography (RIP) using inductive belts, which measure magnetic flux changes to reflect breathing effort [35]. This setup is more wearable and better suited for long-term monitoring, particularly in dynamic environments, with reduced sensitivity to body position. In summary, while the three-channel fusion yields the best detection performance, real-world applications require balancing accuracy with usability and comfort. For example, in home or mobile settings, thoracoabdominal signals may be preferable for their comfort and stability, whereas in clinical contexts where detection precision is essential, incorporating nasal airflow can enhance SDB detection. Therefore, input channel selection should be guided by specific use cases and detection goals.

### 4.6. Limitations

Despite promising results, this study has several limitations, which are as follows:

- Incomplete representation of clinical variability in training data: The current framework is trained primarily on event-containing segments. Including more event-free data could improve robustness and reduce false positives.
- Indirect identification of PLMS: PLMS detection relies on identifying LM and SDB events, followed by the exclusion of RRLM based on AASM guidelines. A dedicated mechanism for RRLM detection has yet to be developed. Future studies may explore a direct approach using respiratory inputs to enhance detection completeness and clinical relevance.
- In addition to model-level limitations, sensor-level considerations are crucial for practical deployment in sleep monitoring. While our study employed standard thoracic, abdominal, nasal airflow, and EMG signals, future work could benefit from more advanced sensing modalities for detecting both SDB and LM. One promising direction is the resonant force sensor based on ionic polymer metal composites (IPMCs), as proposed by Bonomo et al. [36]. These flexible sensors can detect millinewton-scale forces with DC response, making them well suited for capturing subtle physiological signals such as leg movements and thoracoabdominal respiration. Moreover, IPMCs can be fabricated into plastic microelectromechanical systems (PMEMS), enabling seamless integration into wearable devices or bedding for unobtrusive, home-based sleep monitoring.

## 5. Conclusions

We present AttenCRF-U, a joint detection framework for LM and SDB events in OSA patients. By integrating multi-scale feature extraction, MHSA, and CRF, AttenCRF-U leverages the temporal associations between LM and SDB to reduce isolated detection errors and improve the distinction between RRLM and PLMS. MHSA captures long-range dependencies, while CRF enhances sequence coherence. The U-Net backbone with multi-scale convolution further improves sensitivity and boundary accuracy across events of varying durations. Experiments on the Huashan dataset demonstrate strong performance, indicating clinical potential.

Beyond model performance, this study provides biomedical engineering insights. The joint modeling of LM and SDB using standard PSG signals, including thoracic, abdominal, nasal airflow, and EMG channels, reflects their physiological coupling and informs the design of multimodal diagnostic systems. This approach supports simplified sensor configurations and improved diagnostic efficiency. Given its compatibility with streaming architectures, AttenCRF-U is also suitable for integration into wearable or home-based sleep monitoring systems.

By jointly detecting LM and SDB events, the proposed framework improves understanding of their interaction, which is clinically relevant in comorbid PLMS and OSA. Notably, it may inform CPAP titration strategies by identifying RRLMs that influence treatment response, thereby supporting individualized OSA management. This study excluded wake epochs to focus on sleep-specific detection, which may limit applicability in home settings with frequent wake–sleep transitions. Future work will incorporate wake periods and explore generalization across populations and broader sleep event types to enable real-time, low-burden assessment and personalized intervention.

## Figures and Tables

**Table 1:** Demographics and PSG parameters of the Huashan dataset (Mean ± SD, [Min, Max]).

Characteristic	Value
Number	125
Gender (M:F)	91:34
Age	57.59 ± 12.31, [19.00, 85.00]
Total sleep time (TST, min)	372.88 ± 55.86, [241.50, 490.00]
Limb movement index (LMI, /h)	80.38 ± 57.90, [20.13, 401.81]
PLMI (/h)	44.62 ± 32.28, [9.20, 170.50]
OSA severity (mild:moderate:severe, N, %)	46:36:43 (36.8%, 28.8%, 34.4%)
AHI (/h)	26.66 ± 21.45, [5.40, 112.60]

**Table 2:** Descriptive statistics of LM and SDB events (duration in seconds).

Event Type	Number	Median (IQR) (s)	Mean ± SD, [Min, Max] (s)
LM	60,102	1.85 (1.20, 2.67)	2.19 ± 1.49, [0.50, 9.97]
SDB	20,102	27.55 (21.07, 35.44)	29.46 ± 11.80, [10.00, 151.32]

**Table 3:** Performance evaluation results on the Huashan dataset.

Event Type	F1	Pr	Re
LM	0.848	0.862	0.834
SDB	0.728	0.738	0.719
Avg	0.788	0.800	0.777

F1: F1 score; Pr: precision; Re: recall; Avg: average metrics. The same abbreviations are used in the subsequent tables.

**Table 4:** Event-level evaluation results on the Huashan dataset.

Event Type	F1	Pr	Re	Onset Error (s)	Offset Error (s)	Duration Error (s)
LM	0.863	0.849	0.878	0.034	−0.051	−0.085
SDB	0.739	0.703	0.779	2.264	−2.202	−4.466

**Table 5:** Point-wise F1 scores of different methods on the Huashan dataset.

Event Type	MSED [20]	Event-Based Modeling [22]	AttenCRF-U
LM	0.691	0.756	0.848
SDB	0.633	0.705	0.728

**Table 6:** Effect of respiratory signal channels on SDB and LM event evaluation.

Abdo	Thor	Nasal	Avg F1	SDB			LM		
				F1	Pr	Re	F1	Pr	Re
🗸			0.739	0.631	0.622	0.641	0.847	0.825	0.871
	🗸		0.732	0.612	0.599	0.627	0.851	0.852	0.850
		🗸	0.775	0.711	0.670	0.759	0.838	0.867	0.812
🗸	🗸		0.764	0.682	0.686	0.677	0.845	0.828	0.863
🗸		🗸	0.779	0.718	0.702	0.736	0.839	0.870	0.809
	🗸	🗸	0.777	0.706	0.706	0.705	0.848	0.815	0.884
🗸	🗸	🗸	0.788	0.728	0.738	0.719	0.848	0.862	0.834

## Data Availability

The data presented in this study are available on request from the corresponding author due to ethical reasons.

## Abbreviations

The following abbreviations are used in this manuscript:

OSA	obstructive sleep apnea
SDB	sleep-disordered breathing
AHI	apnea–hypopnea index
LM	leg movement
PLMS	periodic limb movements during sleep
PLMI	PLMS index
AASM	American Academy of Sleep Medicine
RRLM	respiratory-related leg movement
MHSA	multi-head self-attention
CRF	conditional random field

## Appendix B. Ablation Study on Convolutional Kernel Combinations

**Table A1:** Ablation study on convolutional kernel combinations in AttenCRF-U.

Kernel Size = 5	Kernel Size = 11	Kernel Size = 17	Avg F1
🗸			0.762
	🗸		0.754
🗸	🗸	🗸	0.695
🗸		🗸	0.773
	🗸		0.763
🗸	🗸	🗸	0.760
			0.788
